# AMPK and SIRT1 activation contribute to inhibition of neuroinflammation by thymoquinone in BV2 microglia

**DOI:** 10.1007/s11010-017-3064-3

**Published:** 2017-05-27

**Authors:** Ravikanth Velagapudi, Abdelmeneim El-Bakoush, Izabela Lepiarz, Folashade Ogunrinade, Olumayokun A. Olajide

**Affiliations:** 1 Department of Pharmacy, School of Applied Sciences, University of Huddersfield, Queensgate, Huddersfield, West Yorkshire HD1 3DH UK; 2 Present Address: Neurobiology Laboratory, National Institute of Environmental Health Sciences, National Institutes of Health, Research Triangle Park, NC USA

**Keywords:** Thymoquinone, AMPKα, ROS, SIRT1, Neuroinflammation

## Abstract

Thymoquinone is a known inhibitor of neuroinflammation. However, the mechanism(s) involved in its action remain largely unknown. In this study, we investigated the roles of cellular reactive oxygen species (ROS), 5′ AMP-activated protein kinase (AMPK) and sirtuin 1 (SIRT1) in the anti-neuroinflammatory activity of thymoquinone. We investigated effects of the compound on ROS generation in LPS-activated microglia using the fluorescent 2′,7′-dichlorofluorescin diacetate (DCFDA)-cellular ROS detection. Immunoblotting was used to detect protein levels of p40^phox^, gp91^phox^, AMPK, LKB1 and SIRT1. Additionally, ELISA and immunofluorescence were used to detect nuclear accumulation of SIRT1. NAD^+^/NADH assay was also performed. The roles of AMPK and SIRT1 in anti-inflammatory activity of thymoquinone were investigated using RNAi and pharmacological inhibition. Our results show that thymoquinone reduced cellular ROS generation, possibly through inhibition of p40^phox^ and gp91^phox^ protein. Treatment of BV2 microglia with thymoquinone also resulted in elevation in the levels of LKB1 and phospho-AMPK proteins. We further observed that thymoquinone reduced cytoplasmic levels and increased nuclear accumulation of SIRT1 protein and increased levels of NAD^+^. Results also show that the anti-inflammatory activity of thymoquinone was abolished when the expressions of AMPK and SIRT1 were suppressed by RNAi or pharmacological antagonists. Pharmacological antagonism of AMPK reversed thymoquinone-induced increase in SIRT1. Taken together, we propose that thymoquinone inhibits cellular ROS generation in LPS-activated BV2 microglia. It is also suggested that activation of both AMPK and NAD^+^/SIRT1 may contribute to the anti-inflammatory, but not antioxidant activity of the compound in BV2 microglia.

## Background

Accumulating evidence suggests that there is a strong link between oxidative stress and inflammation. An unbalanced redox state is known to contribute to the pathogenesis of inflammatory conditions, including ageing [1]. Furthermore, it is now widely accepted that inflammation triggers the generation of elevated levels of cellular reactive oxygen species that cause cellular oxidative damage [2]. On the other hand, inflammatory cells respond to oxidative stress by releasing various NF-κB-mediated pro-inflammatory mediators [3]. Oxidative stress has also been linked to neuroinflammation. Accumulating evidence indicates that reactive oxygen species (ROS) produced by the microglia have a significant impact on adjacent neurons, as well as modulating microglial activity [4]. It has been shown that the activated microglia M1 phenotype is associated with elevated levels of NADPH oxidase (NOX)-dependent ROS generation [5]. Consequently, oxidative stress is now recognised as an important contributor to neuroinflammation, and its resultant neuronal damage in neurodegenerative disorders.

Adenosine monophosphate-activated protein kinase (AMPK) is a well-known sensor of energy balance by responding to ATP-depleting processes such as cellular stress [6]. Recent evidence now suggests that AMPK regulates inflammatory responses [7]. AMPK has been shown to be critical for inducing macrophages from a pro-inflammatory to anti-inflammatory phenotype during inflammation [8]. AMPK activation therefore appears to be a potential strategy for inhibiting inflammation.

Sirtuin 1 (SIRT1) is a member of the sirtuin family which has been linked to cellular processes, including neuroinflammation. SIRT1 is responsible for deacetylation of transcription factors, including p65 subunit of NF-κB during inflammation [9–14]. Due to its negative modulatory effect on inflammation, activation of SIRT1 appears to be critical to achieving anti-inflammatory activity.

**Fig. 1:**
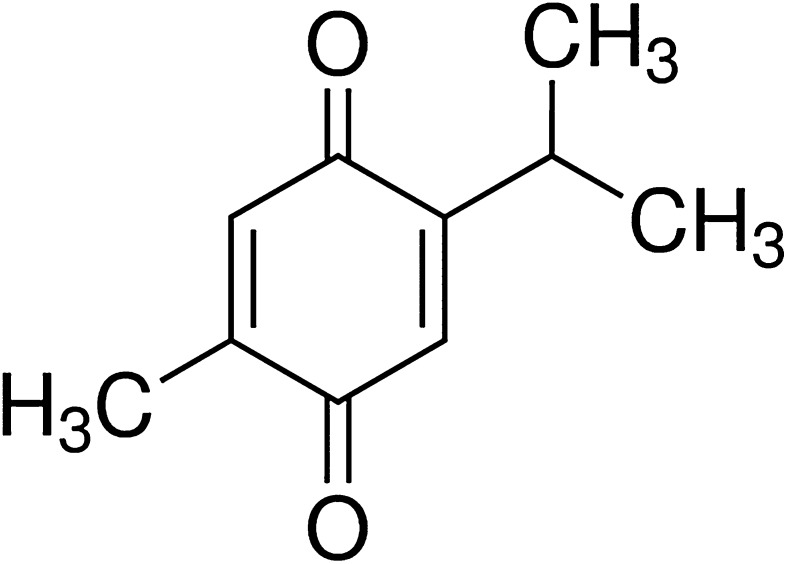
Structure of thymoquinone

Thymoquinone (Fig. 1) is the main constituent of the oil obtained from the seeds of *Nigella sativa* (black cumin seed oil), and has been widely reported to produce anti-inflammatory activity [15–18]. This compound has been shown to inhibit neuroinflammation in mix glial cells [19] and BV2 microglia [20–22].

In this study, we report that thymoquinone activation of both AMPK and SIRT contribute to inhibition of neuroinflammation and cellular ROS by thymoquinone in BV2 microglia.

## Methods

### Cell culture

BV2 mouse microglia cell line ICLC ATL03001 (Interlab Cell Line Collection, Banca Bilogica e Cell Factory, Italy) was maintained in RPMI1640 medium with 10% fetal bovine serum (FBS) (Sigma), 2 mM l-glutamine (Sigma), 100 U/mL penicillin and 100 mg/mL streptomycin (Sigma) in a 5% CO_2_ incubator.

### Intracellular cellular ROS production

Determination of intracellular reactive oxygen species (ROS) levels in BV2 microglia was performed using the fluorescent 2′,7′-dichlorofluorescin diacetate DCFDA-cellular reactive oxygen species detection assay kit (Abcam). Briefly, BV2 microglia were incubated with 10 µM DCFDA for 30 min at 37 °C. After removal of excess DCFDA, the cells were washed and then exposed to LPS (100 ng/mL) for 4 h at 37 °C in the presence or absence of thymoquinone (2.5–10 µM). Intracellular production of ROS was measured by the fluorescence detection of dichlorofluorescein (DCF) as the oxidised product of DCFH on a microplate reader with an excitation wavelength of 485 nm and emission wavelength of 535 nm.

### Immunoblotting

For Western blotting, 20–40 μg of total protein from cell samples was subjected to SDS-PAGE under reducing conditions. Proteins were then transferred onto polyvinylidene fluoride (PVDF) membranes (Millipore). The membranes were blocked for 1 h at room temperature and then incubated overnight at 4 °C with primary antibodies. Primary antibodies used were rabbit anti-SIRT1 (Santa Cruz), rabbit anti-phospho-AMPKα (Santa Cruz), rabbit anti-total AMPKα (Santa Cruz), rabbit anti-LKB1 (Santa Cruz) and rabbit anti-actin (Sigma). Primary antibodies were diluted in Tris-buffered saline (TBS), containing 0.1% Tween 20 (TBS-T) and 1 or 5% BSA. Membranes were incubated with the primary antibody overnight at 4 °C. After extensive washing (three times for 15 min each in TBS-T), proteins were detected by incubation with Alexa Fluor 680 goat anti-rabbit secondary antibody (1:10,000; Life Technologies) at room temperature for 1 h. Detection was done using a LICOR Odyssey Imager. All Western blot experiments were carried out at least three times.

### SIRT1 ELISA

BV2 microglia were seeded in a 6-well plate for 48 h. Thereafter, cells were treated with 2.5–10 µM thymoquinone for 24 h. Following treatment, nuclear extracts were prepared and analysed for levels or SIRT1 using a mouse SIRT1 ELISA kit (Abcam), according to the manufacturer’s instructions.

### NAD^+^/NADH quantification

BV2 microglia were seeded in a 6-well plate and treated with thymoquinone (2.5–10 µM) for 24 h. Cell lysates were collected with 400 μL of NADH/NAD extraction buffer (Abcam). Quantification of NADH and NAD, and their ratio was carried out with a colorimetric NAD/NADH assay kit (Abcam), according to the manufacturer’s instructions.

### RNA interference

Small interfering RNA (siRNA) targeted at mouse AMPK (Santa Cruz Biotechnology) was used to knockout AMPK. BV2 cells were cultured and incubated at 37 °C in a 5% CO_2_ incubator until 70–80% confluent. Cells were then seeded out in a 6-well plate at a density of 1 × 10^6^ cells/well. AMPK siRNA duplex (10 µM) were diluted in Opti-MEM^®^ medium (Thermo Scientific). Lipofectamine^®^ RNAiMAX transfection reagent (Thermo Scientific) was also diluted in Opti-MEM^®^ medium. Thereafter, the diluted siRNA was added to diluted lipofectamine^®^ RNAiMAX reagent (1:1 ratio). The siRNA-lipid complex was then incubated at room temperature for 5 min. The complex was thereafter added to cells and incubated for a further 24 h. Following transfection, media was changed in transfected cells to complete media and incubated for a further 18 h. Effects of thymoquinone (10 µM) on NO, PGE2, TNFα, IL-1β, IL-6 and ROS production in LPS-stimulated control siRNA and AMPK siRNA-transfected BV2 cells were then determined. Similar procedures were used to transfect BV2 cells with mouse SIRT1 siRNA (Santa Cruz Biotechnology), prior to experiments to evaluate effects of thymoquinone (10 µM) on NO, PGE_2_, TNFα, IL-1β, IL-6 and ROS production in LPS-stimulated BV2 cells.

### Evaluation of the effects of AMPK inhibitor on the anti-inflammatory and antioxidant activities of thymoquinone in LPS-activated BV2 microglia

We further investigated whether pharmacological inhibition of AMPK with compound C (Sigma) would affect anti-inflammatory and antioxidant activities of thymoquinone in LPS-activated microglia. Cultured BV2 microglia were treated with compound C (10 µM). One hour later, cells were treated with thymoquinone (10 µM) prior to stimulation with LPS (100 ng/mL) for a further 24 h. Culture supernatants were analysed for levels of TNFα, IL-6, IL1β, NO, PGE_2_. Generation of cellular ROS was determined using the DCFDA method.

### Evaluation of the effects of SIRT1 inhibitor on the anti-inflammatory and antioxidant activities of thymoquinone in LPS-activated BV2 microglia

Pharmacological inhibition of SIRT1 using EX527 (Tocris) was used to determine whether SIRT1 is required for the anti-inflammatory and antioxidant actions of thymoquinone in the microglia. BV2 cells were treated with EX527 (1 µM) followed by thymoquinone (10 µM) prior to stimulation with LPS (100 ng/mL) for 24 h. Culture supernatants were analysed for levels of TNFα (Biolegend, UK), IL-6 (Biolegend, UK), IL1β (Biolegend, UK), NO (Promega, UK), PGE_2_ (Arbor Assays, USA). Generation of cellular ROS was determined using the DCFDA method (Abcam, UK).

### Determination of SIRT-1 activation by thymoquinone in the presence of AMPK inhibitor

We were also interested in the role played by AMPK in the activation of SIRT1 by thymoquinone. Cultured BV2 microglia were therefore treated with thymoquinone (10 µM) in the presence and absence of the AMPK inhibitor, compound (10 µM). Thereafter, protein levels of nuclear SIRT1 were determined using immunoblotting and ELISA. Also, levels of NAD^+^/NADH were quantified in cell extracts.

### Statistical analysis

Values of all experiments were represented as a mean ± SEM of at least 3 experiments. Values were compared using one-way ANOVA followed by a post hoc Student Newman–Keuls test.

## Results

### Generation of NADPH oxidase (NOX)-dependent cellular reactive oxygen species (ROS) in LPS-activated BV2 microglia was inhibited by thymoquinone

**Fig. 2:**
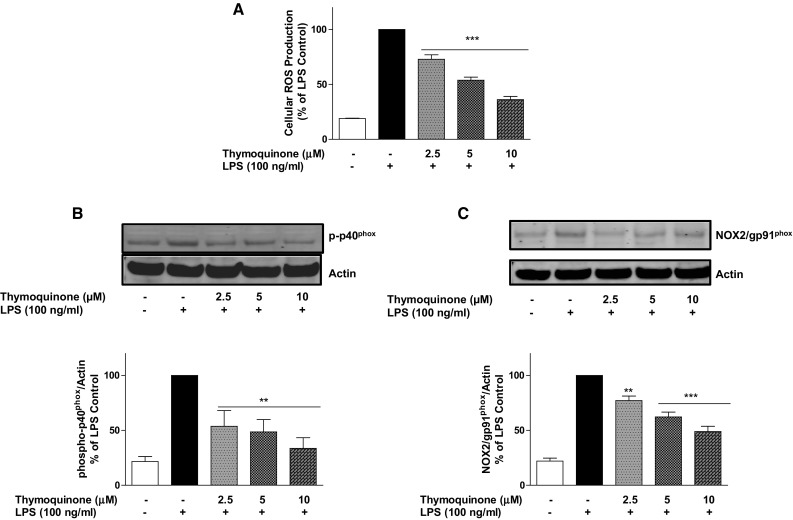
Thymoquinone inhibits NADPH oxidase-mediated ROS generation in LPS-activated BV2 microglia. **a** BV2 cells were treated with vehicle or thymoquinone (2.5–10 µM) for 30 min prior to LPS stimulation for 24 h. ROS generation was measured in live cells by the fluorescence detection of dichlorofluorescein. (Mean ± SEM; ***p < 0.001; one-way ANOVA with post hoc Student Newman–Keuls test). **b** BV2 cells were treated with vehicle or thymoquinone (2.5–10 µM) for 30 min prior to LPS stimulation for 24 h. Cell lysates were analysed using immunoblotting for p-p40^phox^ and actin. Representative blots and densitometric analyses of three independent experiments are shown (Mean ± SEM; **p < 0.01; one-way ANOVA with post hoc Student Newman–Keuls test). **c** BV2 cells were treated with vehicle or thymoquinone (2.5–10 µM) for 30 min prior to LPS stimulation for 24 h. Membrane extracts were analysed using immunoblotting for gp91^phox^ and actin. Representative blots and densitometric analyses of three independent experiments are shown (Mean ± SEM; **p < 0.01, ***p < 0.001; one-way with ANOVA post hoc Student Newman–Keuls test)

Generation of intracellular ROS is a major component of LPS-induced neuroinflammation. We therefore investigated the effect of thymoquinone on LPS-induced generation of ROS in BV2 microglia. Following a 24-h stimulation of microglia with LPS, there was a marked generation of intracellular ROS (Fig. 2a). On treating cells with thymoquinone (2.5, 5 and 10 µM), we observed a significant (*p* < 0.001) and concentration-dependent attenuation of intracellular ROS generation, suggesting that thymoquinone is antioxidant in LPS-activated microglia.

Following our observation that thymoquinone produced antioxidant activity against LPS-induced ROS generation, we were interested in establishing whether this effect was mediated by targeting the NADPH oxidase (NOX) enzymes. To investigate this, we carried out western blotting for the cytoplasmic p-p40^phox^ and membrane-bound gp91^phox^ NOXs in BV2 microglia stimulated with LPS in the presence of thymoquinone. Stimulation of BV2 cells for 24 h resulted in elevation of both p-p40^phox^ and gp91^phox^ proteins (Fig. 2b, c). Treatment of cells with thymoquinone (2.5–10 µM) prior to LPS, resulted in significant reduction of both NADPH oxidases.

### Thymoquinone activates LKB-1/AMPKα in BV2 microglia

**Fig. 3:**
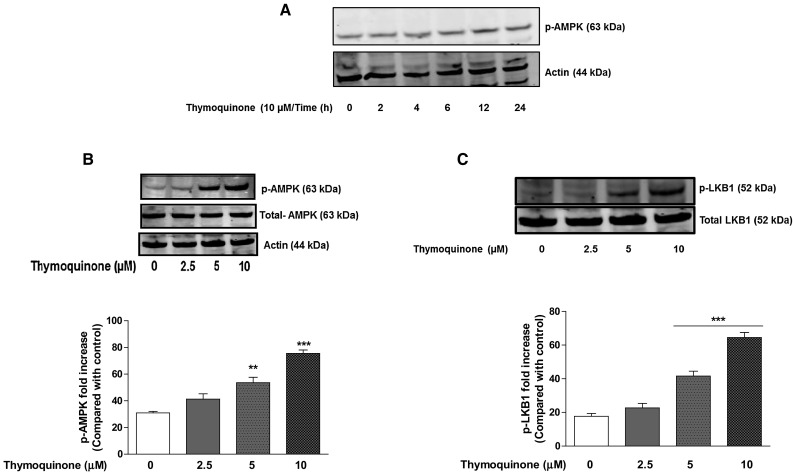
Thymoquinone activates AMPKα (**a**, **b**) and its upstream kinase LKB1 (**c**). BV2 microglia were treated with thymoquinone (2.5–10 µM) for 12 h. Cell lysates were analysed using immunoblotting for phospho-AMPKα and total AMPKα (**a**), LKB1 and actin (**b**). Representative blots and densitometric analyses of three independent experiments are shown (Mean ± SEM; **p < 0.01, ***p < 0.001; one-way ANOVA with ANOVA post hoc Student Newman–Keuls test)

It has been suggested that AMPK contributes to the antioxidant mechanisms by stimulating Nrf2 signalling in immune cells such as RAW 264.7 macrophages [23]. Zimmermann et al. have also shown that activation of AMPK enhances Nrf2/HO1 signalling. We therefore aimed to evaluate the effects of thymoquinone on AMPK and its upstream kinase LKB-1 in BV2 microglia [24]. Preliminary immunoblotting to determine time-point of AMPK activation by thymoquinone (10 µM) shows that maximum activation was detected at 12 h (Fig. 3a). Western blotting experiments show that treatment with thymoquinone (2.5 µM) for 12 h produced a slight increase in the levels of phosphorylated AMPKα. On increasing the concentration of thymoquinone to 5 and 10 µM, there was ~1.7 and ~2.3-fold elevation in the levels of the phosphorylated AMPKα, respectively (Fig. 3b). LKB-1 is a kinase that is known to directly phosphorylate and activate AMPK. Therefore, we evaluated the effect of thymoquinone treatment on LKB-1 protein in BV2 microglia. Our experiments show that incubating BV2 microglia with thymoquinone (5 and 10 µM) for 12 h resulted in an increase in LKB-1 protein in a similar fashion as AMPKα (Fig. 3c).

### AMPK siRNA transfection and compound C treatment reversed anti-inflammatory effect of thymoquinone in LPS-activated BV2 microglia

**Fig. 4:**
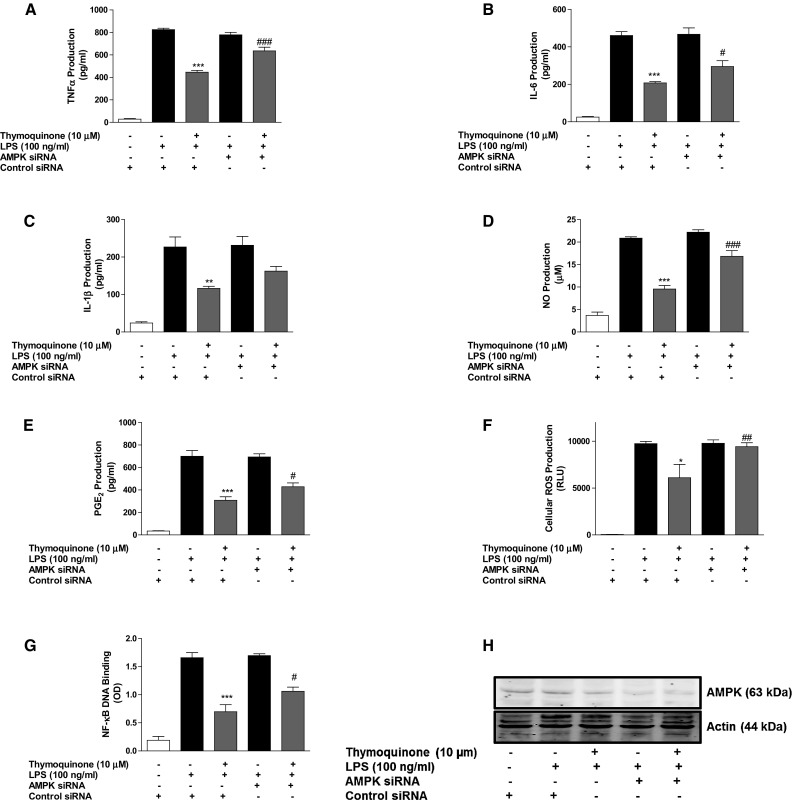
Inhibition of neuroinflammation by thymoquinone is dependent on AMPKα. Control siRNA- and AMPKα siRNA-transfected BV2 cells were pre-treated with thymoquinone (10 μM) prior to stimulation with LPS (100 ng/ml) for 24 h. Cells were analysed for TNFα (**a**), IL-6 (**b**), IL-1β (**c**), nitrite (**d**) PGE_2_ (**e**) and ROS (**f**). In **g** nuclear extracts from cells were added to 96-well plates to which an oligonucleotide containing the NF-κB consensus site (5′-GGGACTTTCC-3′) has been immobilised, followed by addition of NF-κB and HRP-conjugated antibodies. Absorbance was read in a microplate reader. Western blot experiments on cell extracts to determine knockout efficiency (**h**). (Mean ± SEM; **p < 0.01, ***p < 0.001 thymoquinone + LPS treatment compared with LPS alone in control siRNA-transfected cells; ^#^ *p* < 0.05, ^###^ *p* < 0.001, thymoquinone + LPS treatment in AMPK siRNA-transfected cells compared with thymoquinone + LPS treatment in control siRNA-transfected cells; one-way ANOVA with ANOVA post hoc Student Newman–Keuls test)

Based on our observation that thymoquinone could activate AMPKα, we became interested in elucidating the role of this activation on the anti-inflammatory activity of the compound in BV2 microglia. AMPKα siRNA-transfected cells were treated with 10 µM thymoquinone, followed by LPS (10 µM) stimulation for 24 h. Analyses of culture supernatants revealed that thymoquinone (10 µM) prevented the release of pro-inflammatory mediators from control siRNA-transfected BV2 microglia (Fig. 4a–e). Similarly, thymoquinone suppressed binding of activated NF-κB to its consensus binding sites on the DNA (Fig. 4f). In AMPKα siRNA-transfected cells however, the ability of thymoquinone to inhibit NF-κB-mediated neuroinflammation was significantly diminished (*p* < 0.05), in comparison with control siRNA-transfected cells (Fig. 4a–f). Similarly, reduction of LPS-induced ROS generation in BV2 cells was significantly reversed when cells were transfected with AMPK siRNA (Fig. 4g). Western blotting analyses show that AMPK protein was efficiently reduced in AMPK siRNA-transfected cells (Fig. 4h).

**Fig. 5:**
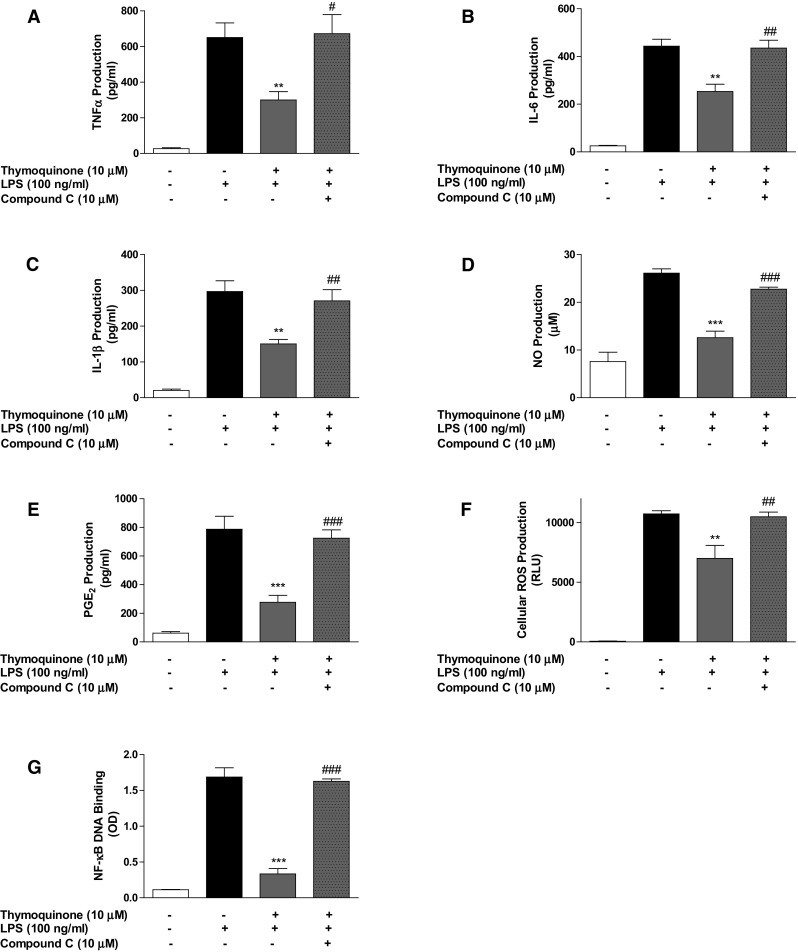
Inhibition of neuroinflammation by thymoquinone was abolished in the presence of compound C. BV2 cells were treated with compound C (10 µM), followed by thymoquinone (10 µM) and LPS (100 ng/ml) for 24 h. Cells were analysed for TNFα (**a**), IL-6 (**b**), IL-1β (**c**), nitrite (**d**), PGE_2_ (**e**) and ROS (**f**). In **g** nuclear extracts from cells were added to 96-well plates to which an oligonucleotide containing the NF-κB consensus site (5′-GGGACTTTCC-3′) has been immobilised, followed by addition of NF-κB and HRP-conjugated antibodies. Absorbance was read in a microplate reader. (Mean ± SEM; **p < 0.01, ***p < 0.001 compared with LPS stimulation; ^#^ *p* < 0.05, ^##^ *p* < 0.01, ^###^ *p* < 0.001, compound C + thymoquinone + LPS treatment compared with thymoquinone + LPS treatment; one-way ANOVA with ANOVA post hoc Student Newman–Keuls test)

To confirm our observations, we treated BV2 cells with a known inhibitor of AMPK, (compound C) prior to thymoquinone treatment and subsequent stimulation with LPS for 24 h. Interestingly, inhibition of inflammatory mediator (TNFα, IL-6, IL-1β, NO, PGE_2_) release as well as NF-κB DNA binding by thymoquinone (10 µM) were significantly (*p* < 0.05) reversed in the presence of compound C (10 µM) (Fig. 5a–f). In Fig. 5g, results show that the effect of thymoquinone on LPS-induced ROS generation in BV2 cells was lost in the presence of compound C.

### Thymoquinone induces NAD^+^-dependent nuclear localisation of SIRT1 in BV2 microglia

**Fig. 6:**
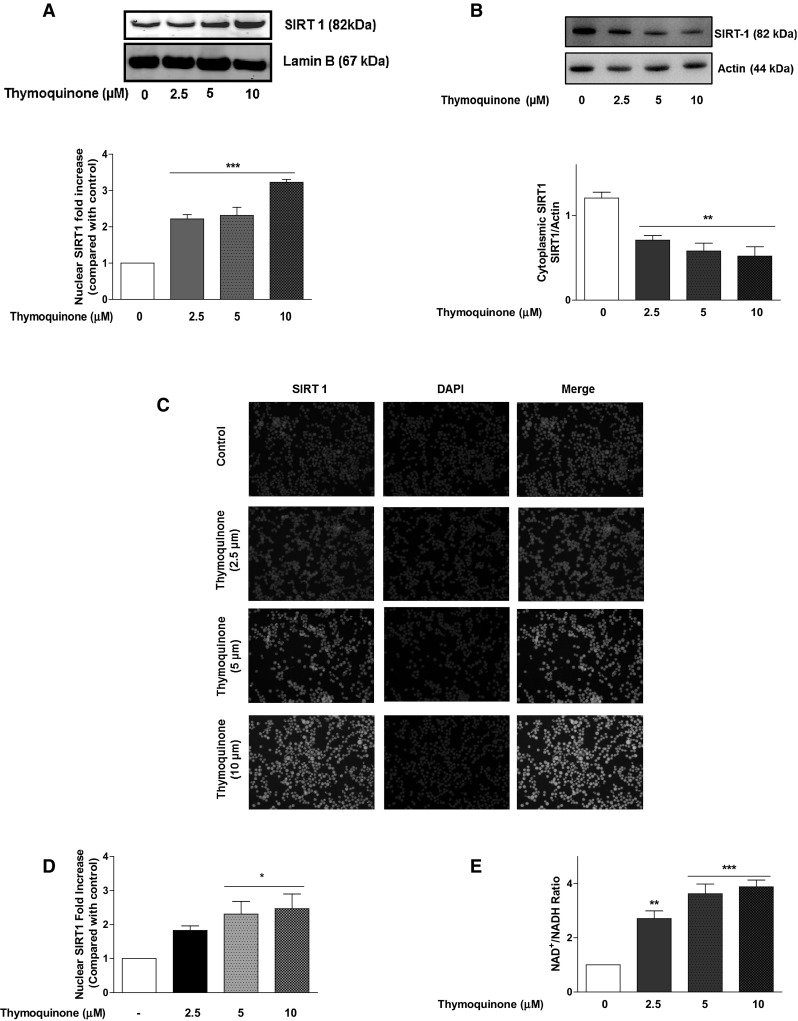
Thymoquinone activates SIRT1 in BV2 microglia. **a** BV2 cells were treated with vehicle or thymoquinone (2.5–10 µM) for 12 h. Nuclear extracts were analysed using immunoblotting for SIRT-1 and lamin B. Representative blots and densitometric analyses of three independent experiments are shown (Mean ± SEM; ***p < 0.001; one-way ANOVA). **b** BV2 cells were treated with vehicle or thymoquinone (2.5–10 µM) for 12 h. Cytoplasmic extracts were analysed using immunoblotting for SIRT-1 and actin. Representative blots and densitometric analyses of three independent experiments are shown (Mean ± SEM; **p < 0.01, ***p < 0.001; one-way ANOVA with ANOVA post hoc Student Newman–Keuls test). **c** Immunofluorescence showing nuclear SIRT1 following treatment of BV2 cells with thymoquinone for 12 h. **d** BV2 cells were treated with vehicle or thymoquinone (2.5–10 µM) for 12 h. Nuclear extracts were analysed using mouse ELISA for SIRT1. (Mean ± SEM; *p < 0.001; one-way ANOVA). **e** Thymoquinone increases levels of NAD^+^ in BV2 microglia. (Mean ± SEM; **p < 0.01, ***p < 0.001; one-way ANOVA with ANOVA post hoc Student Newman–Keuls test)

It is now established that SIRT1 deacetylates p65, resulting in the inhibition of the transcriptional ability of NF-κB [11]. Based on our earlier observation that thymoquinone attenuated LPS-induced p65 acetylation in BV2 microglia, we wanted to find out whether the compound could also be activating SIRT1. Immunoblotting results in Fig. 6a shows that treating BV2 microglia with thymoquinone (2.5–10 µM) for 24 h resulted in significant (*p* < 0.001) accumulation of SIRT1 protein in the nucleus. This was shown to correspond to a concentration-dependent reduction in levels of this sirtuin from the cytoplasm (Fig. 6b). Immunofluorescence data (Fig. 6c) show an increase in SIRT1 immunostaining in BV2 microglia treated with thymoquinone (5 and 10 µM). Similar results were obtained in a mouse SIRT1 ELISA, which shows ~1, ~1.3 and ~1.5-fold increase in SIRT1 in cells treated with 2.5, 5 and 10 µM thymoquinone, respectively (Fig. 6d).

We also investigated the effect of thymoquinone on NAD^+^/NADH ratio in BV2 microglia, and showed that treatment with the compound resulted in statistically significant (*p* < 0.01) and concentration-dependent increase in levels of NAD^+^ (Fig. 6e).

### Transfection of BV2 microglia with SIRT1 siRNA and treatment with EX527 reversed anti-inflammatory action of thymoquinone

**Fig. 7:**
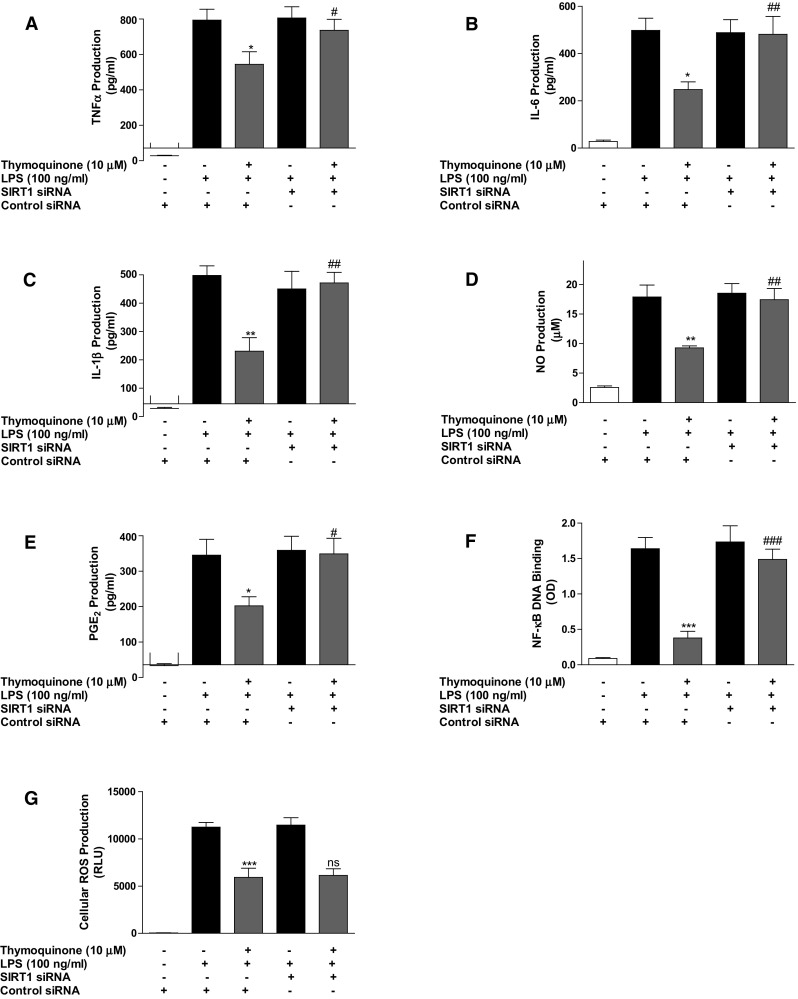
Inhibition of neuroinflammation by thymoquinone is dependent on SIRT1. Control siRNA- and SIRT1 siRNA-transfected BV2 cells were pre-treated with thymoquinone (10 μM) prior to stimulation with LPS (100 ng/ml) for 24 h. Culture supernatants were analysed for TNFα (**a**), IL-6 (**b**), IL-1β (**c**), nitrite (**d**) and PGE_2_ (**e**). In **f** nuclear extracts from cells were added to 96-well plates to which an oligonucleotide containing the NF-κB consensus site (5′-GGGACTTTCC-3′) has been immobilised, followed by addition of NF-κB and HRP-conjugated antibodies. Absorbance was read in a microplate reader. Reduction of cellular ROS production in LPS-stimulated microglia is not dependent on SIRT1 (**g**). (Mean ± SEM; **p < 0.01, ***p < 0.001 thymoquinone +LPS treatment compared with LPS (alone) in control siRNA-transfected cells; ^#^ *p* < 0.05, ^###^ *p* < 0.001, thymoquinone + LPS treatment in SIRT1 siRNA-transfected cells compared with thymoquinone + LPS treatment in control siRNA-transfected cells; one-way ANOVA)

Following observations suggesting that thymoquinone inhibits acetylation of p65 during neuroinflammation as well as activating SIRT1, we were interested in investigating whether direct activation of SIRT1 contributes to the anti-inflammatory action of the compound. Results in Fig. 7a–e show that anti-inflammatory effects of thymoquinone on the production of TNFα, IL-6, IL-1β, nitrite and PGE2 were significantly (*p* < 0.05) abolished following transfection of LPS-activated BV2 microglia with mouse SIRT1 siRNA. Similarly, inhibition of NF-κB DNA binding by the compound was diminished in the presence of SIRT1 siRNA (Fig. 7f). Interestingly, we observed that the ability of the compound to reduce ROS production following LPS stimulation was unaffected by SIRT1 siRNA transfection (Fig. 7g).

**Fig. 8:**
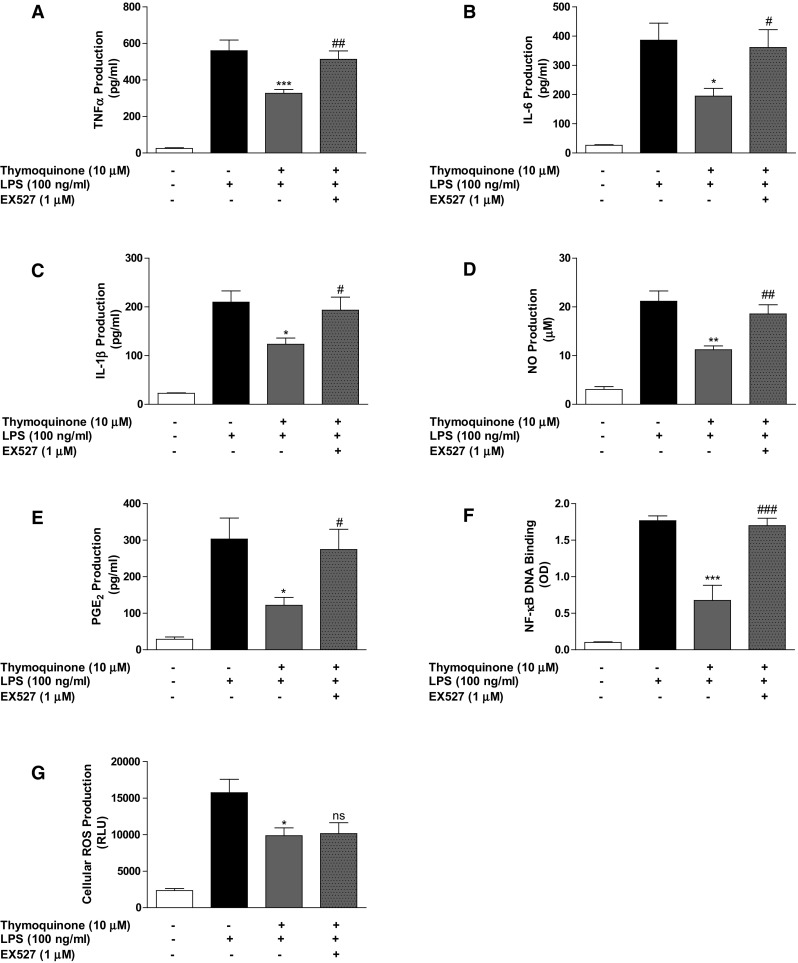
Inhibition of neuroinflammation by thymoquinone was abolished in the presence of EX527. BV2 cells were treated with EX527 (1 µM), followed by thymoquinone (10 µM) and LPS (100 ng/ml) for 24 h. Culture supernatants were collected and analysed for TNFα (**a**), IL-6 (**b**), IL-1β (**c**), nitrite (**d**) and PGE_2_ (**e**). In **f** nuclear extracts from cells were added to 96-well plates to which an oligonucleotide containing the NF-κB consensus site (5′-GGGACTTTCC-3′) has been immobilised, followed by addition of NF-κB and HRP-conjugated antibodies. Absorbance was read in a microplate reader. Reduction of cellular ROS production by thymoquinone was not abolished in the presence of EX527 (G). (Mean ± SEM; **p < 0.01, ***p < 0.001 compared with LPS stimulation; ^#^ *p* < 0.05, ^##^ *p* < 0.01, ^###^ *p* < 0.001, EX527 + thymoquinone + LPS treatment compared with thymoquinone + LPS treatment; one-way ANOVA)

We proceeded to carry out experiments on the role of pharmacological inhibition of SIRT1 on anti-inflammatory effect of thymoquinone by pre-treating BV2 cells with EX527 (1 µM) prior to thymoquinone treatment (10 µM), and followed by stimulation with LPS. Results in Fig. 8a–f show that NF-κB-mediated anti-inflammatory effects of thymoquinone on LPS-induced neuroinflammation were diminished when SIRT1 was inhibited by EX527. However, EX527 treatment did not interfere with antioxidant action of thymoquinone on LPS-induced ROS generation (Fig. 8g).

### Thymoquinone activates SIRT1 through AMPK-dependent mechanisms

**Fig. 9:**
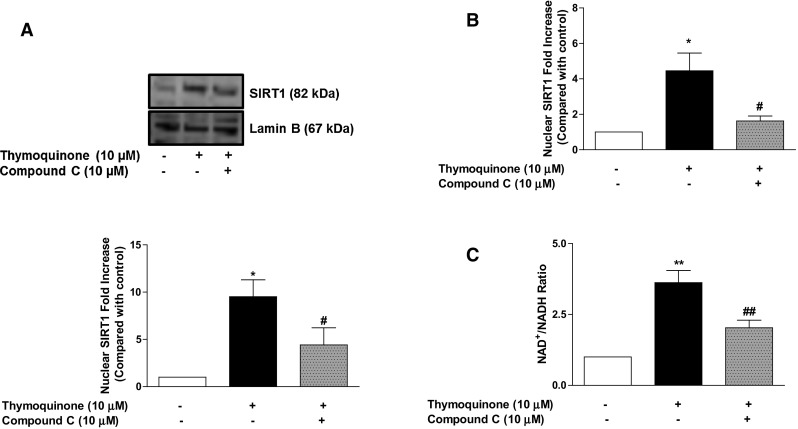
Activation of NAD^+^-dependent SIRT1 by thymoquinone was abolished in the presence of compound C. BV2 cells were treated with compound C (10 µM), followed by thymoquinone (10 µM) for 24 h. Cell extracts were analysed for nuclear SIRT1 (**a**, **b**) and NAD^+^/NADH (**c**). Data and densitometric analyses of three independent experiments are shown (Mean ± SEM; *p < 0.05, **p < 0.01; one-way ANOVA)

Treatment of BV2 microglia with thymoquinone (10 µM) resulted in a significant (*p* < 0.05) increase in nuclear accumulation of SIRT1 (Fig. 9a, b). However, this effect was lost when the cells were exposed to compound C (10 µM) prior to treatment with thymoquinone. Similarly, there was increase in NAD^+^ levels produced as a result of thymoquinone treatment was diminished in the presence of compound C (10 µM) (Fig. 9c).

## Discussion

Activation of microglia during neuroinflammation is known to result in the generation of ROS by a process that is facilitated by the NADPH oxidase enzymes [25, 26]. Furthermore, excessive production of ROS through NADPH oxidase is known to be responsible for neuroinflammation and neurodegeneration [27]. We show that thymoquinone produced a marked reduction in ROS generation induced by LPS stimulation in BV2 microglia, suggesting that the compound is able to attenuate ROS generation in response to neuroinflammation. It is known that activated microglia cells are associated with elevated levels of NADPH oxidase (NOX)-dependent ROS generation [5]. NOX is a multi-subunit enzyme complex which transfers electrons from NADPH to molecular oxygen, forming O_2_^−^. Under resting conditions, the different subunits of this complex are localised in the cytosol (p40^phox^, p47^phox^ and p67^phox^) and in the cell membrane (p22^phox^ and gp91^phox^). However, following stimulation of the microglia, the complex is assembled in the plasma membrane [27]. Once we established that thymoquinone reduced ROS generation in activated microglia, we then proceeded to show that thymoquinone produced a reduction in elevated levels of p-p40^phox^ and gp91^phox^ proteins in LPS-stimulated microglia, suggesting that an inhibition of these NOX enzymes contribute to the inhibitory effects of the compound on ROS generation during neuroinflammation. Consistent with our findings, thymoquinone was shown in other studies to reduce ROS generation in inflammation [28, 29].

There have been suggestions in the scientific literature linking AMPK activation to inhibition of inflammation [30]. Our results reveal that thymoquinone treatment increased the levels of phosphorylated AMPKα and its upstream kinase LKB1 in the microglia, suggesting that this compound activates the LKB1/AMPKα signalling pathway in the microglia. Thymoquinone has earlier been reported to produce anti-inflammatory effect as well as enhancing the phosphorylation AMPK and LKB1 in a mouse model of fibrosis [31]. In another study reported by Yang et al., the compound activated AMPK in hepatic stellate cells [32]. To our knowledge, this is the first report showing that thymoquinone activates AMPK in the microglia.

Based on reports showing inhibition of neuroinflammation by thymoquinone [20–22], and our observation on the inhibition of cellular ROS generation in LPS-activated microglia, we then asked whether there was a relationship between these activities and AMPK activation by the compound. Interestingly, it was revealed that transfection with AMPKα siRNA as well as pre-treatment with AMPK inhibitor (Compound C) resulted in the loss of anti-inflammatory and ROS inhibitory activities of thymoquinone in LPS-activated BV2 microglia. Studies have linked AMPK to inflammation and redox mechanisms. For example, studies reported by Lin et al. [33] and Tsai et al. [7] suggest that lycopene and caffeic acid phenethyl ester (CAPE) produce anti-neuroinflammatory effect through AMPKα-dependent mechanisms. Similar observations have been reported for berberine [34], ginseng [35] and paeonol [36]. We suggest that activation of AMPK possibly contributes to inhibition of neuroinflammation by thymoquinone, which sheds more light on the mechanisms involved in the activity of this compound.

It has been reported that AMPK activation can promote expression of genes which are involved in antioxidant defence mechanisms [37]. Also, AMPK activation has been shown to increase NAD^+^ levels, resulting in SIRT1 activation and deacetylation-induced activation of targets such as FOXO3a [38]. Interestingly, AMPK activation has been linked to nuclear accumulation of the antioxidant transcription factor Nrf2 [24, 39]. Studies have also shown that upregulation of AMPK abolished the levels of LPS-enhanced NOX-derived ROS in human brain microvascular endothelial cells [40]. Our results showing that inhibition of ROS by thymoquinone is reversed in the presence of AMPK gene knockout and compound C seem to suggest that activation of AMPK is likely involved reducing LPS-induced ROS generation, and contributes to the antioxidant activity of the compound.

It is now known that inhibition of neuroinflammation could be achieved through processes involved in the deacetylation of p65. The NAD^+^-dependent deacetylase SIRT1 is known to deacetylate p65, resulting in the inhibition of the transcriptional ability of NF-κB [11], and the resulting appearance of pro-inflammatory genes. We therefore became interested in establishing whether this could be linked to a direct effect of the compound on SIRT1. Interestingly, thymoquinone treatment induced significant accumulation of SIRT1 protein in the nucleus of BV2 microglia, while inducing its disappearance from the cytoplasm. The compound also increased the levels of NAD^+^ in the cells, suggesting an NAD-dependent activation of SIRT1. These results suggest that thymoquinone could be blocking LPS-induced p65 acetylation through a direct activation of SIRT1, which then deacetylates the protein. The natural SIRT1 activator, resveratrol, has been similarly shown to target NF-κB activation by promoting deacetylation of RelA/p65 in Aβ-stimulated microglia [12]. Resveratrol has also been shown to inhibit inflammatory response through attenuation of LPS-induced production of NO, PGE_2_, iNOS, COX-2, TNFα, IL-1β, as well as inhibition of NF-κB activation in BV2 microglia [41]. We further established that SIRT1 gene knockout and EX527 treatment resulted in the loss of anti-inflammatory but not antioxidant activity of thymoquinone in LPS-activated BV2 microglia. These observations further confirm that thymoquinone activates SIRT1 in the nucleus, resulting in the deacetylation and nuclear export of p65 NF-κB. This results in suppression of transcription of NF-κB-controlled pro-inflammatory genes such as TNFα, IL-6, IL-1β, as well as COX-2 and iNOS. Our results are similar to those obtained in a study reported by Yang et al., which shows that thymoquinone enhanced SIRT1 protein levels in human hepatic stellate cells [32].

**Fig. 10:**
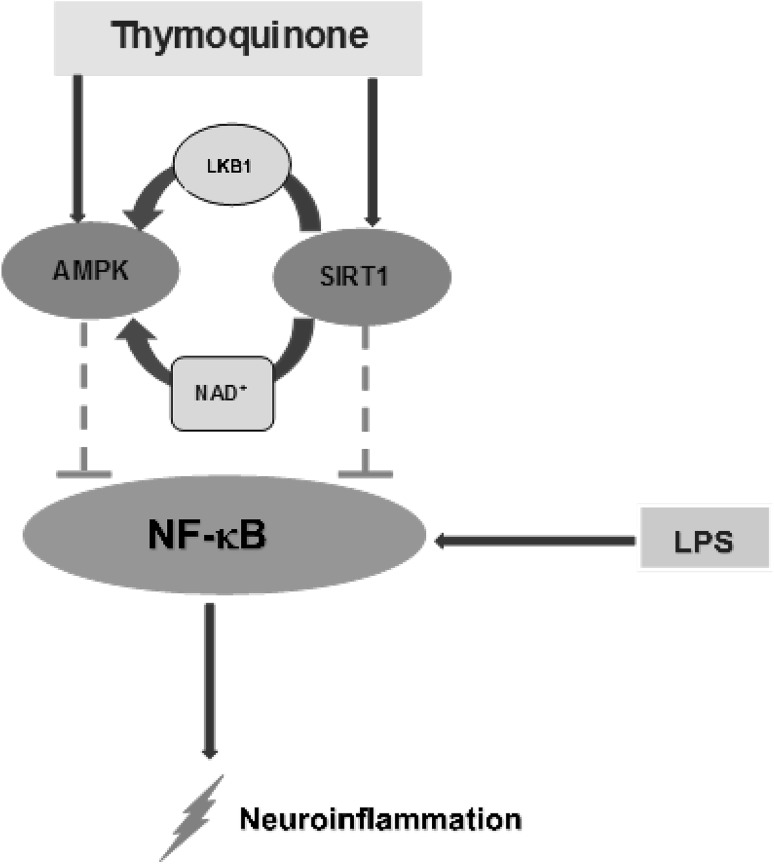
Inhibition of neuroinflammation by thymoquinone involves activation of AMPK and SIRT1

We further showed that NAD^+^-dependent activation of SIRT1 by thymoquinone requires AMPK. This further suggests a possible interaction between SIRT1 and AMPK in the activity of thymoquinone in BV2 microglia. This interaction may account for the anti-inflammatory activity of the compound in LPS-activated microglia (Fig. 10).

This study has established that thymoquinone inhibits NOX-mediated ROS generation and activates both AMPK and SIRT1 in BV2 microglia. These actions are proposed to contribute to the anti-inflammatory activity of the compound in activated microglia. Further studies are required to determine whether this compound activates SIRT1 in neurons to produce neuroprotective effects.
